# The glucocerebrosidase mutations and uric acid levels in Parkinson’s disease: A 3-years investigation of a potential biomarker”

**DOI:** 10.1016/j.prdoa.2022.100177

**Published:** 2022-12-17

**Authors:** Mehrdad Mozafar, Sina Kazemian, Elahe Hoseini, Mohammad Mohammadi, Rojina Alimoghadam, Mahan Shafie, Mahsa Mayeli, The Parkinson's Progression Markers Initiative

**Affiliations:** a NeuroTRACT Association, Students’ Scientific Research Center, Tehran University of Medical Sciences, Tehran, Iran; b Cardiac Primary Prevention Research Center, Cardiovascular Diseases Research Institute, Tehran University of Medical Sciences, Tehran, Iran; c Iranian Center of Neurological Research, Imam Khomeini Hospital Complex, Tehran, Iran; d School of Medicine, Tehran University of Medical Sciences, Tehran, Iran; e Medical Imaging Department, AMT School, Isfahan Medical Sciences University, Isfahan, Iran

**Keywords:** Glucocerebrosidase mutations, Uric acid levels, DaT scan specific binding ratio, Parkinson’s disease

## Abstract

**Background:**

Blood uric acid level indicates an emerging biomarker in Parkinson's disease (PD). This study aimed to evaluate longitudinal uric acid levels among different kinds of glucocerebrosidase (GBA) mutations and to compare it among sporadic PD, genetic cohort Parkinson's disease (GENPD), genetic cohort unaffected (GENUN), and healthy control (HC) patients.

**Methods:**

We conducted a study on 654 individuals from the Parkinson's progression markers initiative (PPMI) database. Baseline characteristics, uric acid levels, movement disorder society unified Parkinson's disease rating scale III (MDS-UPDRS III), Hoehn and Yahr Parkinson stage (H&Y stage), and DaT scan specific binding ratio (SBR) data were obtained. Different GBA mutations were collected and categorized into three groups. Longitudinal measurements of uric acid and MDS-UPDRS III score were evaluated during 3-years of follow-up.

**Result:**

GENPD cohort exhibited a greater MDS-UPDRS III score, H&Y stage, and lower SBR in the right caudate, left caudate, and right putamen compared to sporadic PD. Baseline uric acid level was similar among all groups and different GBA variants. After adjustment for age, sex, and body mass index, the uric acid level was significantly lower in the GENPD group than in HC during year 2 (P-value: 0.009). No significant longitudinal differences were detected for the MDS-UPDRS III score and three groups of GBA mutations.

**Conclusion:**

This is the first study to assess uric acid levels and MDS-UPDRS III scores among different GBA mutation variants within 3 years of follow-up. We found similar clinical characteristics among different subtypes of GBA mutations.

## 1 Introduction

Parkinson's disease (PD) is the second most frequent neurodegenerative disorder worldwide, with a more than twofold increase in global burden over the past generation [1] and the number of cases is estimated to increase twofold by 2030 [2]. PD is characterized by several debilitating features, including bradykinesia, tremor, depression, anxiety, and cognitive impairments, decreasing patients' quality of life [3], [4]. Although the etiology remains obscure in most cases, a broad spectrum of monogenic forms has been proposed for PD over the past two decades [5] and it is agreed that the complexity of PD stems from the interaction of genetic and environmental factors, comorbidities, and aging [6].

Mutations in key genes of the endosomal/lysosomal system such as leucine-rich repeat kinase 2 (LRRK2), glucocerebrosidase (GBA), and adenosine three phosphates (ATP) are associated with an increased risk of PD by increasing α-synuclein protein accumulation and Lewy bodies formation [7]. GBA gene encodes lysosomal glucocerebrosidase enzyme (GCase), which cracks open sphingolipids into glucose and ceramide [8]. It is found that homozygote mutations of GBA can cause Gaucher disease, while heterozygous vectors are at risk for developing PD [9]. In previous studies, GBA1 mutation was associated with early-onset, the relatively symmetrical onset of limb symptoms, and faster motor and cognitive impairment progression in PD patients; however, the association between different GBA mutation subtypes are less studied [10], [11]. It is noteworthy that former research has suggested that variants of GBA could be associated with faster progression of motor symptoms, accompanied by accelerated conversion to cognitive impairment and dementia. Therefore, genetic modifiers, such as GBA variants, could account for some of the clinical heterogeneity observed in PD [12].

Uric acid (UA) is an endogenous antioxidant agent suggested to have a protective role in PD [13]. Previous studies have postulated the role of higher UA levels, which reduced the risk of both the development and progression of sporadic PD [14], [15]. For instance, a recent study reported UA as a marker linked to LRRK2-PD [13], while another study proposed UA as a disease progression biomarker for GBA-PD [10].

Nevertheless, there is a lack of evidence about the association between UA level and different GBA variant mutations regarding clinical and imaging findings. This study aims to investigate baseline and 3-year longitudinal differences in serum UA levels among patients with sporadic PD, GBA mutation PD, GBA mutation carriers, and healthy controls enrolled in the Parkinson's progression markers initiative (PPMI) study. In addition, we will compare these findings in different GBA mutation subtypes.

## 2 Methods

### 2.1 Ethical considerations

The study population and participants' data were obtained from the PPMI database (https://www.ppmi-info.org/data). The present study protocol corresponds to the 2013 Helsinki declaration. According to the study protocol, informed consent was obtained from all recruited participants [16].

### 2.2 Study design and participants

PPMI is a multicenter study that aims to identify PD-related biomarkers by providing an international database of clinical, genetic, biospecimen, and imaging data from 21 clinical sites [16]. We conducted a study on 291 sporadic PD, 65 genetic cohort Parkinson's disease (GENPD), 166 genetic cohort unaffected (GENUN), and 132 healthy control (HC) individuals using the PPMI database (accessed December 2021). PPMI enrolled all patients with PD (including sporadic PD, GENPD, GENUN) who met the following criteria: individuals aged 30 years or older with a diagnosis of PD for 2 years or less at the screening visit, patients must have at least two of the following symptoms: resting tremor, bradykinesia, rigidity (must have either resting tremor or bradykinesia); OR either asymmetric resting tremor or asymmetric bradykinesia, Hoehn and Yahr Parkinson stage (H&Y stage) I or II at baseline, confirmation that participant is eligible based on screening dopamine transporter scan (DaT) scan imaging. Exclusion criteria were: 1) those who currently receive levodopa, dopamine agonists, monoamine oxidase inhibitors (MAO-B inhibitors) (e.g., selegiline, rasagiline), amantadine, or another PD medication, 2) patients who have taken MAO-B inhibitors, or amantadine within 60 days of baseline visit, 3) individuals who have taken levodopa or dopamine agonists before baseline visit for more than a total of 90 days, and 4) patients with any other medical conditions and lab abnormalities, 5) individuals with lack of key information in the database records. Additionally, among GENPD and GENUN cohorts, those who had genetic mutations other than GBA mutations (including LRRK2 and synuclein alpha (SNCA)) were also excluded from the study. We have categorized GBA variant IDs into three groups, including group 1: heterozygote N409S mutation (N409S); group 2: homozygote N409S mutation (N409S/N409S), and group 3: other GBA variants IDs like L29Afs 18, L483P and IVS2 + 1G > A. Compound heterozygote mutations (N409S/E365K, N409S/R83C) were excluded from the study to attain more exquisite results.

### 2.3 Definitions and data collection

According to PPMI, the GENPD group was defined as individuals with symptomatic genetic PD, while the GENUN group was defined as individuals with asymptomatic genetic PD (LRRK2, SNCA, or GBA mutations). HC was defined as clinically normal 30 years old or older individuals with no DaT scan abnormality and no first-degree family history of PD [16].

Demographic data, including age, gender, body mass index (BMI), and UA level, were obtained for all the cohorts mentioned above. We further obtained data regarding the movement disorder society unified Parkinson's disease rating scale III (MDS-UPDRS III), H&Y stage, and DaT scan specific binding ratio (SBR) for PD and GENPD cohort. Regarding MDS-UPDRS III score in the GENPD group, screening data were not available for 28 individuals; thus, the baseline data were used instead. It is noteworthy that all motor assessments were conducted Off-medication, based on PPMI's manual [16]. All of the PPMI participants underwent a DaT scan which is a specific type of single-photon emission computed tomography (SPECT) imaging, at PPMI imaging centers 4 ± 0.5 h after injection of 3 to 5 mCi of 123I-ioflupane. Imaging results were analyzed according to the imaging technical operations manual (https://ppmi-info.org/). Imaging data were presented as the regional SBR of the striatal subregion (putamen, caudate nucleus).

### 2.4 Serum UA measurement

Routine laboratory tests were performed at the first visit only for new enrollments who were being screened for PD. A central laboratory conducted the analyses and utilized consistent normal ranges to enable common interpretations. Venous whole blood was collected in vacutainers. All samples for laboratory analysis were collected, prepared, labeled, and shipped according to the laboratory's requirements as detailed in the PPMI's lab manual. No more than 60 ml of blood was drawn at either the screening or baseline visit. Whole blood (about 10 ml), serum (about 30 ml), and plasma (about 10 ml) were collected to conduct metabolomics, genetic and other research analyses. The research blood samples were collected in a fasted state (i.e., a minimum of 8 h since the last meal/food intake) to ensure the quality of samples for future analyses. If fasting was not possible, then participants were advised to eat a low-lipid diet. Participants did not receive any individual results of research analysis or testing conducted on the biological samples. A further detailed description of the biological samples of PPMI can be found in the PPMI Biologics Manual.

### 2.5 Genetic assessments

The DNA from the blood or saliva samples stored at Massachusetts General Hospital (MGH) Neurogenetics DNA/Biochemical Diagnostic Laboratory was used for genetic testing. The subjects signed an appropriate consent form to be categorized as either GENPD Participants or GENUN Participants. The site study coordinator completed the GBA Screening form and sent the form to the GCC study coordinator. The stored samples for GBA N370S were sent in batches. The GCC study coordinator requested MGH that stored DNA samples be screened for GBA N370S. The GCC study coordinator will ship the saliva sample to Massachusetts General Hospital (MGH) for LRRK2 and/or GBA mutation testing. Operations Manual PPMI Genetics Study is available for further information (https://www.ppmi-info.org/study-design/research-documents-and-sops).

### 2.6 Statistical analyses

Categorical variables are presented as numbers (percentage), and numerical variables are reported as mean ± standard deviation or median [interqueartile range] throughout the study. Kolmogorov Smirnoff test and Shapiro Wilk test were used to assess normality in numerical variables when sample sizes are above 30 and below 30, respectively. The independent T-test and one-way ANOVA tests were used to analyze numerical variables with normal distribution, while Kruskal-Wallis and Mann-Whitney U tests were used for non-parametrical variables. Categorical variables were compared using Fisher's exact test and the chi-squared test. Longitudinal changes of UA and MDS-UPDRS III were evaluated with repeated measures ANOVA. Also, Pearson correlation and Spearman correlation were calculated for comparison between baseline UA and MDS-UPDRS III for GENPD and sporadic PD, respectively. To perform a linear mixed effects analysis of the relationship between UA and motor progression, as fixed effects, we entered UA, age, sex, and BMI (without interaction term) into the model. As the random effect, we had intercepts for subjects. P-values were obtained by likelihood ratio tests of the full model with the effect in question against the model without the effect in question. All statistical analyses were performed using the statistical package for the social sciences (SPSS) 21.0 software. Statistical significance was set at P-value < 0.05.

## 3 Results

A total number of 654 individuals enrolled in our final analysis. Patients were categorized into 4 groups: sporadic PD (N = 291, 44.5 %), GENPD (N = 65, 9.9 %), GENUN (N = 166, 25.4 %), and HC (N = 132, 20.2 %). Overall female/male ratio was 375/279, and the mean age and BMI were 61.27 ± 9.21 years and 27.05 ± 4.76 kg/m^2^, respectively. Demographic and baseline characteristics in each group are presented in Table.1. There was no difference in age and BMI between the groups. The female-to-male ratio was significantly higher in GENUN patients compared to other groups. The Baseline UA level was similar among these groups. At the same time, disease duration, MDS-UPDRS III score, and H&Y stage were significantly higher in the GENPD cohort compared to patients with sporadic PD. Moreover, the GENPD group had a significantly lower SBR in the right caudate, left caudate and right putamen compared with the sporadic PD group.

**Table 1:** Baseline and clinical characteristics in sporadic PD, GENPD, GENUN, and healthy control groups.

Characteristics†		Sporadic PD (N = 291)	GENPD (N = 65)	GENUN (N = 166)	Healthy control (N = 132)	P-value*
Age (years)		61.0 ± 9.40	63.29 ± 8.86	61.85 ± 6.87	60.15 ± 11.21	0.111
Sex	Female	101 (34.7 %)	29 (44.6 %)	99 (59.6 %)	50 (37.9 %)	**<0.001**
	Male	190 (65.3 %)	36 (55.4 %)	67 (40.4 %)	82 (62.1 %)	
BMI (kg/m^2^)		26.98 ± 4.64	26.60 ± 4.55	27.77 ± 5.22	26.52 ± 4.46	0.222
Disease duration (months)		6.57 ± 6.67	38.75 ± 25.88	N/R	N/R	**<0.001**
GBA mutation variants	N409S	N/R	52 (80.0 %)	148 (89.2 %)	N/R	0.759
	N409S/N409S		5 (7.7 %)	12 (7.2 %)		
	Others		8 (12.3 %)	6 (3.6 %)		
Baseline uric acid (mg/dL)		5.32 ± 1.33	5.04 ± 1.27	5.26 ± 1.37	5.41 ± 1.33	0.327
MDS-UPDRS III		19 [13–26]	25 [18–36]	N/R	N/R	**<0.001**
H&Y stage		2 [1–2]	2 [2–2]	N/R	N/R	**<0.001**
DaT scan SBR	Right caudate	1.97 ± 0.56	1.72 ± 0.72	N/R	N/R	**0.015**
	Left caudate	1.97 ± 0.54	1.74 ± 0.71			**0.001**
	Right putamen	0.83 ± 0.33	0.72 ± 0.45			**0.001**
	Left putamen	0.79 ± 0.82	0.73 ± 0.40			0.088

† Data are presented as mean ± standard deviation, number (%), or median [interquartile range].

*Statistically significant P-values are bolded.

Abbreviations: BMI: body mass index, DaT scan: dopamine transporter scan, GBA: glucocerebrosidase, GENPD: genetic cohort Parkinson's disease, GENUN: genetic cohort unaffected, H&Y stage: Hoehn and Yahr Parkinson stage, MDS-UPDRS III: movement disorder society unified Parkinson’s disease rating scale III, N/R: not reported, PD: Parkinson’s disease, SBR: specific binding ratio.

The demographic and clinical characteristics of the three different subtypes of GBA mutations are shown in Table 2. Among patients with GENPD, disease duration was significantly higher in patients with mutations other than N409S (P-value: 0.040), with a mean difference of 18.73 and 35.9 months from patients with N409S (heterozygote mutation) and N409/N409 (homozygote mutation), respectively. Our results showed that age, baseline UA level, MDS-UPDRS III, H&Y stage, and DaT scan SBR were similar across different mutations of GBA.

**Table 2:** Comparing demographic and clinical characteristics between three groups of mutations.

Characteristics†		N409S (N = 200)	N409S/N409S (N = 17)	Others (N = 14)	P-value*
Age (years)	GENUN	62.21 ± 6.67	59.11 ± 8.12	58.36 ± 8.24	0.588
	GENPD	63.86 ± 8.47	61.30 ± 11.95	60.83 ± 10.05	0.146
Disease duration (months)	GENUN	N/R	N/R	N/R	**0.040**
	GENPD	37.77 ± 26.01	20.60 ± 13.59	56.50 ± 22.08	
Baseline uric acid (mg/dl)	GENUN	5.33 ± 1.40	4.78 ± 1.05	4.56 ± 0.90	0.186
	GENPD	5.04 ± 1.27	5.56 ± 1.58	4.75 ± 1.12	0.545
MDS-UPDRS III	GENUN	N/R	N/R	N/R	0.868
	GENPD	25 [19–34.25]	35 [11–44.5]	24 [18.25–44.75]	
H&Y stage	GENPD	N/A	N/A	N/A	0.427
	GENUN	2 [2–2]	2 [2–2.5]	2 [1.25–2.75]	
DaT scan SBR					
GENUN	Right Caudate	N/R	N/R	N/R	N/R
	Left Caudate				
	Right Putamen				
	Left Putamen				
GENPD	Right Caudate	1.78 ± 0.76	1.70 ± 0.40	1.31 ± 0.51	0.345
	Left Caudate	1.72 ± 0.76	1.63 ± 0.30	1.50 ± 0.48	0.914
	Right Putamen	0.74 ± 0.48	0.78 ± 0.38	0.48 ± 0.13	0.227
	Left Putamen	0.75 ± 0.43	0.67 ± 0.18	0.68 ± 0.27	0.800

† Data are presented as mean ± standard deviation, number (%), or median [interquartile range].

* Statistically significant P-values are bolded.

Abbreviations: DaT scan: dopamine transporter scan, GENPD: genetic cohort Parkinson's disease, GENUN: genetic cohort unaffected, H&Y stage: Hoehn and Yahr Parkinson stage, MDS-UPDRS III: movement disorder society unified Parkinson’s disease rating scale III, N/R: not reported, SBR: specific binding ratio.

**Fig. 1:**
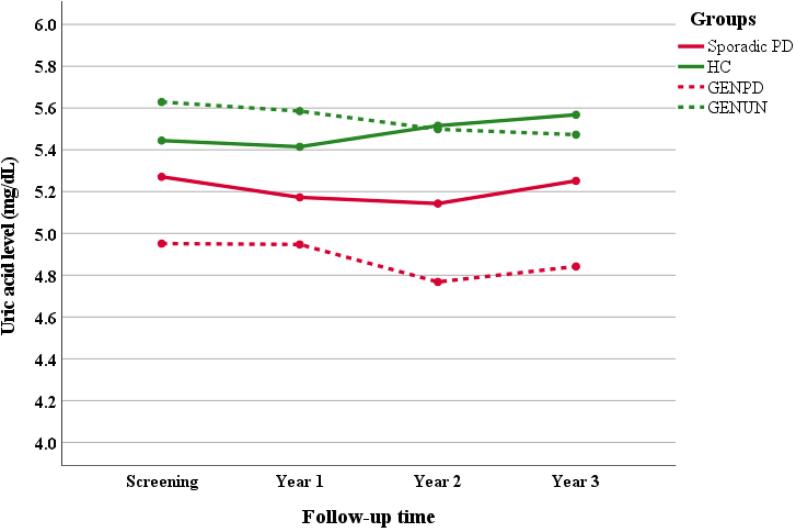
3-year longitudinal measurements of mean uric acid level among sporadic PD, healthy control, GENPD, and GENUN groups. GENPD: genetic cohort Parkinson's disease, GENUN: genetic cohort unaffected, HC: healthy control, PD: Parkinson’s disease.

Out of 654 cases, we selected 368 patients with available 1-, 2-, and 3-year UA and MDS-UPDRS III score follow-up data. After adjustment for age, sex and BMI, we compared 3-year longitudinal UA levels and MDS-UPDRS III score across these groups. Longitudinal measurements of serum UA levels are presented in Fig. 1. There was no between-group difference in UA levels during each follow-up period except in year 2 (P-value: 0.034). Further post-hoc analysis revealed that the UA level was significantly lower in the GENPD group compared to HC during year 2 (mean difference: 0.87 mg/dL, P-value: 0.009). We observed no effect of “Time” or “Time*Group” interaction on the UA level during follow-up (P-value: 0.231 and P-value: 0.519, respectively). Moreover, as we evaluated the longitudinal 3-year UA levels among N409S and N409S/N409S mutations, no effect of “Time” or“ Time*Group” interaction was observed among different groups of GBA mutations.

**Fig. 2:**
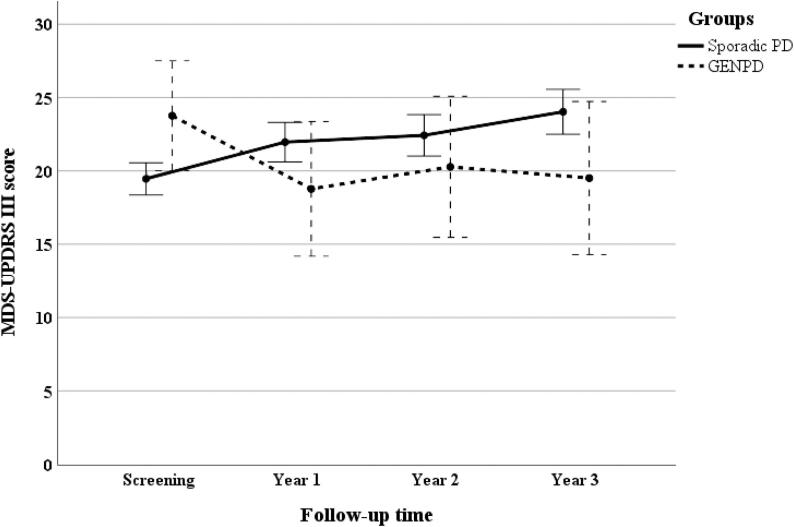
3-year longitudinal measurements of mean MDS-UPDRS III score among sporadic PD and GENPD groups. Error bars represent a confidence interval of 95%. GENPD: genetic cohort Parkinson's disease, GENUN: genetic cohort unaffected, HC: healthy control, MDS-UPDRS III: movement disorder society unified Parkinson’s disease rating scale III, PD: Parkinson’s disease.

Longitudinal measurements of the MDS-UPDRS III score during the 3-year follow-up are presented in Fig. 2. MDS-UPDRS III score was greater during screening time in patients with GENPD, but it decreased and remained steady during the follow-up period. On the other hand, patients with sporadic PD followed a steadily increasing trend of MDS-UPDRS III score during 3-years of follow-up. We observed no statistical difference among sporadic PD and GENPD groups in each follow-up period. There was a significant “Time*Group” interaction but no “Time” effect on MDS-UPDRS III score (P-value: 0.004 and P-value: 0.806, respectively). No significant correlations were found between baseline UA level and MDS-UPDRS III score at baseline (P-value: 0.16, P-value: 0.64), year 1 (P-value: 0.54, P-value: 0.88), year 2 (P-value: 0.14, P-value: 0.53) and year 3 (P-value: 0.15, P-value: 0.63) among patients with sporadic PD and GENPD groups, respectively.

Eventually, we performed a linear mixed effects model to explore the longitudinal relationship between UA and motor progression in the sporadic PD and GENPD groups. Our analysis showed that UA did not significantly affect UPDRS in sporadic PD group (χ2 (1) = 0.348, p = 0.555) and in GENPD group (χ2 (1) = 0.543, p = 0.461). There was no significant group interaction (sporadic PD vs GENPD) regarding the effect of UA on UPDRS (χ2 (3) = 1.714, p = 0.634).

## 4 Discussion

UA has been introduced as a novel biomarker for predicting PD, associated with disease progression and severity [17], [18]. Nevertheless, there is scarce evidence regarding UA changes in patients carrying PD-associated mutations such as GBA or LRRK2 gene mutations [10], [19]. In this study, we found similar baseline and 3-year longitudinal UA levels and MDS-UPDRS III scores among patients with sporadic PD, GENPD, GENUN, and HC except for lower UA levels in GENPD patients compared to HC during year 2. Furthermore, clinical characteristics were similar among different subtypes of GBA mutation. It is noteworthy that the higher MDS-UPDRS III in GENPD compared to sporadic PD at baseline is congruent with former research, suggesting more severe initial symptoms in those patients with the genetic type of PD [20].

UA has powerful antioxidant properties by scavenging the reactive oxygen species, which provide neuroprotective properties against PD [21]. UA may also induce the accumulation of SNCA and autophagy activation [22]. It has been discovered that PD patients have lower UA serum levels than healthy individuals, and this difference is more considerable in males than in females [23]. In an in vivo study conducted by Huang et al. on rats with PD, UA showed neuroprotective properties for dopaminergic neurons by modulation of oxidative stress and neuroinflammation through increasing protein expression of NF-E2-related factor 2 (Nrf2) and three Nrf2-responsive genes [24]. Moreover, A study conducted by Zhong et al. showed that the UA/creatinine ratio and UA levels in early and medium-stage PD patients were significantly higher in patients with advanced-stage PD, which introduces UA as a biomarker for disease severity [18].

Previous studies have reported that higher levels of serum UA have been associated with a lower risk of PD and a milder course of disease [25], [26], [27]. Conversely, some studies reported similar longitudinal UA measurements compared to sporadic PD patients with HCs [17]. In this study, we observed similar baseline UA levels among the four groups (sporadic PD, GENPD, GENUN, and HC), which is in line with previous findings. The mentioned previous research [16] suggested that its relatively small sample size and short duration of follow-up could have affected the findings, which can be similarly attributed to our study. To properly evaluate the potential utility of UA as a progression marker and in distinguishing various mutations in PD, future studies with greater sample sizes and longer follow-ups are recommended. Furthermore, there was no difference in UA levels between different subtypes of GBA mutations. The role of UA was highlighted in patients with PD's associated genetic mutations. UA was investigated as a possible biomarker of PD risk oscillation in carriers of LRRK2 mutation [28], [29]. It is found that individuals who developed PD had significantly lower UA levels than those with non-manifesting LRRK2 mutation, and a 2 mg/dL increase in UA levels reduced the feasibility of having PD by about 50 % [29], [30].

Another important group of PD patients is those harboring GBA gene mutations. There is a lack of evidence regarding UA changes in patients with GBA mutations [10]. In this study, we observed no 3-year longitudinal differences in serum UA levels between patients with sporadic PD, GENPD, GENUN, and HC except for significantly lower UA levels in patients with GENPD compared to HC (mean difference: 0.87 mg/dL) during the second year. After evaluating different types of GBA mutation, we observed no 3-year longitudinal differences between UA levels and GBA mutation subtypes. In a study by Simuni et al. over 184 GBA mutation carriers, there was no difference in serum UA levels between asymptomatic GBA1 carriers and HCs [19].

We also evaluated the MDS-UPDRS III score during the follow-up period. There was significant“ Time*Group” interaction after investigating 3-year longitudinal measurements. Despite patients with GENPD, patients with sporadic PD had a steady increasing trend in their MDS-UPDRS III score during the follow-up, proving faster disease progression and reduced motor function in patients with sporadic PD. A study conducted by Huang evaluating the impact of UA on motor and non-motor symptoms in PD patients showed that higher serum UA levels are associated with the more benign tremor dominant motor subtype than the postural instability gait disorder mixed phenotypes. Moreover, there was lower fatigue in PD patients with higher UA levels, which might be attributed to the anti-oxidative effects of UA [31]. We observed no correlation between the UA level and MDS-UPDRS III score among sporadic PD and GENPD patients during the 3-year follow-up period. It is to be mentioned that, unlike our research, in this previous study, the GENUN group was not taken into consideration. Besides, our findings provide the results of a 3-year follow-up, while the previous work only evaluated the subjects for 2 years. Eventually, additional mutations and subgroup analyses were performed in the present study to further enrich our investigation.

This is the first study investigating UA levels and MDS-UPDRS III scores among different GBA mutation variants within 3-years of follow-up. Nevertheless, we emphasize that it has some limitations. First, according to the PPMI inclusion protocol, the majority of patients carry mainly the N409S mutation, which is considered to be mild [32]. Consequently, our study participants might not entirely represent the total GBA-PD population. Second, the small sample size in some subgroups may have influenced the study results, and further multi-central studies with larger sample sizes are warranted. Additionally, as none motor symptoms in PD are relatively recently suggested to predict the disease way before the onset of motor symptoms, future studies are recommended to thoroughly investigate the potential associations of UA with these symptoms and in predicting their progression compared to the motor symptoms investigated in this research.

## 5 Conclusion

In conclusion, we found similar baseline and 3-year longitudinal UA levels and MDS-UPDRS III scores among patients with sporadic PD, GENPD, GENUN, and HC except for lower UA levels in GENPD patients compared to HC during year 2. Furthermore, clinical characteristics were similar among different subtypes of GBA mutation. As the protective nature of higher UA in PD has been recently investigated, our work confirms the previous findings in terms of lower UA in GENPD compared to controls at the one-time point. Considering differences in various mutations, although our research yielded insignificant differences between the four investigated groups, future studies with larger sample sizes and longer follow-up durations are required to further evaluate potential differences. Additionally, investigating the association between serum UA and other carrying pathogenic mutations is recommended, which may reveal novel therapeutic options in PD patients.

## CRediT authorship contribution statement

**Mehrdad Mozafar:** Conceptualization, Methodology, Software. **Sina Kazemian:** Conceptualization, Methodology, Software. **Elahe Hoseini:** Writing – review & editing. **Mohammad Mohammadi:** Writing – review & editing. **Rojina Alimoghadam:** Writing – review & editing. **Mahan Shafie:** Writing – review & editing. **Mahsa Mayeli:** Conceptualization, Project administration, Writing – review & editing.

## Declaration of Competing Interest

The authors declare that they have no known competing financial interests or personal relationships that could have appeared to influence the work reported in this paper.
